# Prognostic and predictive value of EGFR in head and neck squamous cell carcinoma

**DOI:** 10.18632/oncotarget.11413

**Published:** 2016-08-19

**Authors:** Paolo Bossi, Carlo Resteghini, Nicholas Paielli, Lisa Licitra, Silvana Pilotti, Federica Perrone

**Affiliations:** ^1^ Head and Neck Cancer Medical Oncology Department, Fondazione IRCCS Istituto Nazionale dei Tumori, Milan, Italy; ^2^ Laboratory of Experimental Molecular Pathology, Department of Pathology, Fondazione IRCCS Istituto Nazionale dei Tumori, Milan, Italy

**Keywords:** EGFR, head and neck squamous cell cancer, prognostic factors, predictive factors, radiotherapy

## Abstract

EGFR is an extensively studied biomarker in head and neck squamous cell carcinoma (HNSCC). In this review, we discuss the prognostic and predictive role of EGFR in HNSCC, focusing on the different molecular alterations in specific treatment modalities such as radiotherapy alone (RT), combination of surgery, RT and chemotherapy (CT), EGFR inhibitors. We considered EGFR at different molecular levels: protein expression, protein activation, gene copy number, polymorphisms, mutation, EGFRvIII expression and EGFR ligand expression.

Considering RT alone, evidence supports the predictive and prognostic role of high EGFR expression only when evaluated by quantitative assays: this may help select the patients who can mostly benefit from accelerated treatment. Conversely, no predictive biomarkers are available when treatment is a combination of surgery, CT and RT. For this combined treatment, several studies indicate that EGFR expression represents a good prognostic parameter only when measured by a “quantitative” or at least semi-quantitative method. With respect to EGFR inhibitors, neither EGFR expression nor increased gene copy number represent prognostic/predictive factors.

If validated, nuclear EGFR, TGFα levels, EGFR phopshorylation and polymorphisms could represent additional prognostic factors in relation to combination of surgery, CT and RT, while EGFR polymorphisms and high amphiregulin levels could have prognostic value in patients treated with EGFR inhibitors.

## INTRODUCTION

Head and neck squamous cell carcinoma (HNSCC) represents the sixth most frequent cancer worldwide with an incidence of 560,000 cases/year and over 350,000 deaths annually [1]. Treatment of locally-advanced HNSCC requires a multidisciplinary approach involving surgery followed by radiation (RT) with or without chemotherapy (CT) or curative RT with concomitant CT or anti-EGFR agents. *TP53* gene status [2, 3] and HPV [4] are the most studied biological markers with known prognostic value. The extensive studies of HPV have paved the way for tailored therapeutic strategies, with the aim of sparing toxicities in HPV-positive tumors and intensifying treatment in HPV-negative cancers.

As observed also for other malignancies, an extensively studied biomarker in HNSCC is the epidermal growth factor receptor (EGFR), a cell surface receptor member of the ErbB family. Activation of EGFR leads to a phosphorylation cascade mediated via tyrosine kinases which works downstream through the PI3K–PTEN–AKT, MAPK, ERK, and Jak/STAT pathways and promotes proliferation, invasion, angiogenesis, and metastatic spread. Evidence of EGFR activity has been reported in HNSCC cell lines, as well as in a high percentage of primary HNSCC [5-7]. Aberrant activation of EGFR signaling in HNSCC may be achieved by several mechanisms, including overexpression of EGFR and its ligands, establishing autocrine/paracrine loops, *EGFR* gene amplification, EGFR mutation/polymorphism and transactivation by other receptor tyrosine kinases (RTKs). The relevance of EGFR pathway in HNSCC led to the successful development of cetuximab in both the curative and palliative settings [8,9] and to the conduction of several trials with other antibodies directed against EGFR, such as panitumumab, zalutumumab and nimotuzumab [10-13], or RTK inhibitors involving downstream EGFR signaling [14, 15].

Several studies have investigated the prognostic and predictive value of EGFR in HNSCC. In this review, we discuss available evidence on this topic, focusing on the different EGFR molecular alterations in tumor tissue, in relation with different treatments and settings.

## SEARCH CRITERIA

To identify the key publications on EGFR prognostic or predictive value in HNSCC, we conducted a comprehensive literature search in the online database Medline. The search was last updated on October 2015 and included only articles in English, with no limitation on the publication date. Articles were selected for inclusion and assigned to each single treatment section, as judged by the Authors.

Clinical outcome was evaluated in terms of clinical response, overall survival (OS), progression-free survival (PFS), disease-free survival (DFS), locoregional control (LRC), locoregional relapse (LRR), locoregional failure (LRF), disease control rate (DCR) or time to treatment failure (TTF), depending on the reported results of considered studies.

A *prognostic* biomarker was defined as any tumor characteristic that informs about cancer outcome. In more detail, a biomarker was defined as prognostic when patients with tumor showing a specific characteristic have different survival than subjects without that specific characteristic, independently from the treatment [16]. A *predictive* biomarker was defined as a tumor characteristic that can be used to predict the tumor response to a specific treatment. In particular, the biomarker is considered predictive if the treatment effect is different for patients with tumor showing a specific characteristic compared with patients without that specific characteristic [16]. Therefore, a predictive biomarker can be evaluated only in head-to-head studies presenting both treated and control arms.

In our analysis we considered EGFR at different cytogenetic/molecular levels: protein expression, protein activation, gene copy number, polymorphisms, mutation, EGFRvIII expression and EGFR ligand expression. From identified papers we retrieved prognostic and predictive information regarding EGFR alterations according to the treatment provided. Treatments were grouped as: radiotherapy alone (RT); combination of surgery, RT and chemotherapy (CT); EGFR inhibitors.

## CLINICAL EVIDENCE

## EGFR PROTEIN EXPRESSION

### Methods of assessment

The expression of EGFR protein has been evaluated by several means (Table 1). EGFR immunohistochemistry (IHC), a relatively easy and inexpensive technique, represents the most frequent option; moreover, tissue microarrays enable multiple samples to be stained at once, favoring investigation of biomarkers in large case series of IHC. However, in available studies, EGFR immunoreactivity was heterogeneously evaluated using different cut-off values and following different criteria for intensity and/or extent of the staining, as well as cytoplasmic and/or membranous staining.

Quantitative *in situ* molecular-based methods have been developed to define EGFR expression by IHC thus avoiding the subjectivity of visual assessment. Another way of determining EGFR expression is the binding assay, a less-used method based on frozen samples processed with radioactive labeled EGF and used to estimate EGFR concentration.

**Table 1:** Main studies on EGFR protein expression as prognostic and predictive factor in HSCC

Study	Population	Treatment	Method	Prognostic value of high EGFR	Predictive value of high EGFR
**Radiotherapy**					
Chang 2008	n=50, glottic SCC	Primary conventional fractionated or hypofractionated RT	IHC assay	Impact on recurrence, TTP	-
Parik 2007	n=123, laryngeal SCC	Primary RT	IHC assay	No impact on OS, LRR	-
Wen 1996	n=68, laryngeal SCC	RT	IHC assay	No impact on OS, RR	-
Nichols 2012	n=75, laryngeal SCC	RT	IHC assay	No impact on OS, LRR	-
Aebersold 2002	n=95, oropharyngeal SCC	primary RT	IHC assay	No impact on OS, DFS, LTC	-
Lassen 2013	n=336, oropharyngeal SCC	primary RT	IHC assay	No impact on OS, DFS, LRC	-
Ryott 2009	n=78, oral tongue SCC	Preoperative RT	IHC assay	No impact on pCR	-
Ang 2002	n=155, HNSCC	primary RT	SAMBA system	Impact on OS, DFS, LRR	-
Chung 2011	n=533, HNSCC	Accelerated or standard fractionated RT	SAMBA system	Impact on OS, LRR, PFS in both arms	No impact on OS, PFS, LRR in accelerated RT arm
Bentzen 2005	n=304 HNSCC	CHART vs conventional fractionated RT	IHC assay	-	Impact on LRC in the CHART arm
Eriksen 2005	n=209, supraglottic larynx SCC	Primary RT, OTT: 9½, 6½ or 5½ weeks	IHC assay	-	Impact on LRC in the arms with OTT 6½ or 5½ weeks
**Surgery, chemotherapy, radiotherapy**					
Ranelletti 2001	Laryngeal SCC	Surgery +/− RT	Binding assay	Impact on OS	-
Dassonville 1993	n=109, HNSCC	CT	Binding assay	Impact on OS, relapse free	-
Magné 2001	n=77, oro and hypopharynx SCC	Non accelerated RT+CT	Binding assay	Impact on OS, TTF	-
Etienne 1999	n=82, advanced HNSCC	Preoperative CT +/− RT	Binding assay	Impact on OS	-
Pivot 2005	n=71, hypo and larynx SCC	Preoperative CT; primary RT	Binding assay	Impact on OS, DFS	-
Almadori 1999	n=140 laryngeal SCC	Surgery +/− RT	Binding assay	Impact on neck node relapse	-
Maiorano 1998	N=100 oral cavity SCC	surgery	IHC assay	Impact on DFS, OS	
Grandis 1998	n=91, HNSCC	Surgery +/− RT +/− CT	SAMBA system	Impact on DFS	-
Psyrri 2005	n=67, oropharyngeal SCC	Primary RT; surgery and RT +/− CT	AQUA	Impact on response (nuclear EGFR), LRR, DFS	-
Pectasides 2011	n=64, HNSCC	Primary RT or surgery + RT	AQUA	Impact on OS	-
Szabo 2011	n=71, HNSCC	Surgery	IHC assay	Impact on OS	-
Kontic 2015	n=185, laryngeal SCC	surgery	IHC assay	Impact on OS	-
Huang 2012	n=160, oral cavity SCC	surgery	IHC assay	Impact on DFS, OS	-
Monteiro 20012	n=67, oral cavity SCC	Surgery +/− RT	IHC assay	Impact on OS, DFS	-
Farhadied 2009	n=106, laryngeal SCC	Surgery +/− RT	IHC assay	Impact on OS; DFS; LRR	-
Laimer 2007	n=109, oral and oropharyngeal SCC	Surgery +/− CT +/−RT	IHC assay	Impact on OS	-
Wheeler 2012	n=154, HNSCC	Surgery +/− RT or CRT	IHC assay	Impact on OS, PFS	-
Lindquist 2012	n=62, oropharyngeal SCC	Preoperative RT +/− surgery	IHC assay	Impact on OS	-
Jiang 2009	Laryngeal SCC	Surgery + RT	IHC assay	No impact on OS LRC	-
Nakata 2011	n=89, oral tongue SCC	Surgery	IHC assay	No impact on DFS and OS	-
Lundberg 2012	n=130, HNSCC	Surgery +/− RT +/− CT	IHC assay	No impact on DFS	-
Ongkeko 2005	n=44, pharynx and larynx SCC	Surgery	IHC assay	No impact on DFS	-
Carracedo 2008	n=47, pharynx and larynx SCC	Surgery	IHC assay	No impact on OS, relapse	-
Won 2012	n=121, oral and oropharyngeal SCC	Surgery +/− RT +/− CT	IHC assay	No impact on RFS	-
Trivedi 2011	n=135, oral SCC	Surgery +/− RT +/− CT	IHC assay	No impact on OS, RFS	-
Shah 2009	n=89, oral SCC	Surgery +/− RT +/− CT	IHC assay	No impact on OS, RFS	-
Diniz.feitas 2007	n=44, oral SCC	surgery	IHC assay	No impact on OS	-
Shiraki 2005	n=140, oral SCC	surgery	IHC assay	No impact on OS	-
Rahimi 2012	n=106, oropharyngeal SCC	IMRT+ CT	IHC assay	No impact on OS, DFS, LRC	-
Szentkuti 2015	n=226, HNSCC	unknown	IHC assay	No impact on OS	-
Keren 2014	Meta-analysis 37 studies	Surgery +/−RT+/−CT	IHC assay	Impact on OS	-
Numico 2010	n=122, HNSCC	CT or surgery +CT	IHC assay	No impact on OS, PFS, LRC	-
Perisanidis 2013	n=113, oropharyngeal SCC	Preoperative CRT + surgery	IHC assay	No impact on OS, response	-
Kumar 2008	n=50, oropharyngeal SCC	Preoperative CT+/−RT	IHC assay	Impact on response to induction CT, OS, DSS	-
Hitt 2005	n=46, HNSCC	Preoperative CT	IHC assay	Impact on DFS and OS	-
Hong 2010	n=270, oropharyngeal SCC	Surgery; RT; surgery + RT	IHC assay	Impact on LRF	-
Reimers 2007	n=80, oropharyngeal SCC	Surgery + RT; CRT	IHC assay	No impact on DFS, OS	-
Young 2011	n=240, HNSCC	CRT	IHC assay	No impact on FFS, OS	-
Shi 2009	n=111, oropharyngeal SCC	RT; CRT	IHC assay	No impact on OS, DFS	-
Kong 2009	n=82, HNSCC	RT; CRT; surgery +/− RT+/−CRT	IHC assay	Impact on OS	-
Vainshtein 2014	n=198, oropharyngeal SCC	CRT	IHC assay	No impact on LRF	-
**EGFR inhibitors**					
Hitt 2012	n=33, recurrent and/or metastatic HNSCC	Paclitaxel + Cetuximab	IHC assay	No impact on response, PFS, OS	-
Tinhofer 2011	n=47, recurrent and/or metastatic HNSCC	Docetaxel + cetuximab	IHC assay	No impact on DCR, PFS, OS	-
Psyrri 2005	n=57, HNSCC	CT +/− cetuximab	IHC assay	No impact on OS, PFS	Impact on response to cetuximab
Ang 2014	n=380 HNSCC	CRT+/− cetuximab	IHC assay	No impact on PFS, OS, LRF	-
Wheeler 2012	n=39, recurrent/metastatic HNSCC	Cisplatin, docetaxel and Cetuximab + RT	IHC assay	Impact on OS	-
Smilek 2012	n=29, HNSCC	RT+ cetuximab	Real time PCR	Impact on pCR	-
Psyrri 2014	n=63, HNSCC	CT+cetuximab+CRT	AQUA	No impact on OS, PFS	-
Burtness 2005	n=117, HNSCC	Cisplatin +/−Cetuximab	IHC assay	-	Impact on response. No impact on PFS, OS
Licitra 2012	n=411, recurrent and/or metastatic HNSCC	Cisplatin or carboplatin, 5FU +/− cetuximab	IHC assay	-	No impact on OS, response, PFS
Basavaraj 2010	n=92, HNSCC	Cisplatin +/− nimotuzumab	IHC assay	Impact on OS	-
Crombet 2004	n=24, HNSCC	RT+nimotuzumab	IHC assay	No impact on OS	-
Rodriguez 2010	N=55, HNSCC	RT +/− nimotuzumab	IHC assay	-	Impact on OS

HNSCC: head and neck squamous cell carcinoma; OS: overall survival; PFS: progression free survival; RT: radiotherapy: CT: chemotherapy: CRT: chemotherapy; IHC: immunohistochemistry; IMRT: intensity modulated RT; RR: recurrence rate: LRR: locoregional relapse; LTC: local tumor control; TTF: time to treatment failure; pCR: pathological complete response; DSS: disease specific survival; RFS: relapse free survival; -: the prognostic or predictive value of high EGFR expression was not investigated.

### 1A) RADIOTHERAPY

#### Prognosis

Four different studies [17-20] investigated EGFR expression, evaluated by IHC in laryngeal SCCs treated exclusively with primary RT, as prognostic factor. All but one of these studies reported no association between high EGFR expression and OS or LRC [18-20].

Similarly, an intense EGFR expression assessed by IHC did not influence LRC, DFS and OS in two different series of oropharyngeal SCC patients treated with primary RT [21, 22], as well as in response of oral tongue SCCs treated with preoperative RT [23].

By contrast, in two HNSCC series not differentiated by subsite and treated with exclusive RT, EGFR expression was a robust prognostic factor only when a “quantitative” EGFR image analysis-based IHC assay was performed. In one study, EGFR expression, quantitatively evaluated by using the SAMBA system, was a strong independent prognostic indicator, capable to improve the estimation of OS, DFS and LRC applied to pretreatment biopsy specimens from patients assigned to the standard therapy arm receiving conventional RT [24]. The same “quantitative” approach was used in a series of HNSCC patients enrolled into a Phase III trial who received accelerated or standard fractionated RT [25]. Regardless of treatment modalities, high EGFR expression was associated with higher LRR (Relative Risk: 1.91, *P*=0.0163) and lower OS (Relative Risk: 1.90, *P*=0.0010). Collectively, these findings show that high EGFR expression assessed by a quantitative method might be a prognostic marker associated with poor outcome of HNSCC patients treated exclusively with RT.

#### Prediction

In a series of HNSCC patients enrolled into a Phase III trial who received accelerated or standard fractionated RT, high EGFR expression (quantitatively assessed) showed a trend as independent determinant of OS [hazard ratio (95% CI for interaction): 0.75 (0.45-1.25)], PFS (hazard ratio (95% CI for interaction): 0.77 (0.45-1.31) and LRR (hazard ratio (95% CI for interaction): 0.88 (0.46-1.71)] in the accelerated arm, although statistical significance was not reached [25]. Once again, IHC analysis performed in pretreatment tumor biopsies from HNSCC patients revealed a beneficial role of high EGFR expression in patients assigned to continuous hyperfractionated accelerated RT (CHART) compared with conventionally-fractionated RT [26]. Particularly, among patients showing high EGFR expression (cells with EGFR membrane staining ≥25%), a significant benefit in 3-year LRC rate was observed in the CHART arm compared with the conventionally-fractionated RT arm. No difference between the two treatment arms was observed in the low EGFR expressing group. However, EGFR expression had no significant effect on OS or distant metastases [26].

Another study suggests the predictive value of EGFR expression in HNSCC patients treated exclusively with RT [27]. Patients with supraglottic larynx SCC were treated with primary RT at the same total dose but with different OTT: 9½ weeks, 6½ weeks or 5½ weeks. Using LRC as endpoint, the results showed that patients whose tumors had high EGFR expression at IHC assay benefitted from a reduction in RT overall treatment time from 6½ to 5½ weeks more than subjects with low EGFR levels.

Collectively, high EGFR expression may be useful for selecting HNSCC patients who will benefit from accelerated RT in terms of better LRC.

### 1B) COMBINATION OF SURGERY, RADIOTHERAPY AND CHEMOTHERAPY

#### Prognosis

Among the studies that used binding assays, some investigated the role of EGFR within organ preservation strategies such as neoadjuvant CT followed by RT or concurrent CT/RT. No correlation between EGFR levels and response to these treatments was disclosed. However, with such technique, most studies reported a positive correlation between high concentrations of EGFR and poorer prognosis [28-32]. The binding assay confirmed also EGFR expression to be an independent prognostic factor of neck node relapse in HNSCC patients undergoing surgical resection ±RT [33].

The first attempts to establish a relationship between EGFR expression measured by IHC and PFS and OS were performed by two independent groups several years ago [34, 35]. In both cases, high EGFR expression was a predictor of reduced DFS and OS at multivariate analysis. However, while Maiorano et al [34] analyzed by EGFR IHC a series of oral cavity SCC treated with surgery and scored samples as positive when presenting at least 10% of either membranous or cytoplasmic stain, Rubin Grandis et al [35] relied on an automated image analysis system (SAMBA) to quantify EGFR expression by IHC in the primary tumor of patients who underwent surgery ±RT and CT.

More recently, the prognostic impact of EGFR protein level measured by quantitative approaches was confirmed by two independent studies which assessed EGFR expression through the AQUA method [36, 37]. In particular, Psyrri et al [36] composed a tissue microarray of primary, non-metastatic oropharyngeal SSC treated with primary external beam RT or gross total surgical resection and postoperative RT (±CT). The AQUA scoring system showed that patients with high cytoplasmic or nuclear EGFR expression were more likely to experience local recurrence. In a tissue microarray composed of HNSCC treated with CTRT, AQUA analysis revealed EGFR protein levels as a strong predictor of patient outcome [37].

On the other hand, discordant results were obtained when EGFR expression was assessed by IHC without a quantitative approach. In most of the studies involving case series of HNSCC treated with surgery ±CT/RT, semi-quantitative criteria were adopted considering both intensity and extent of membranous staining. However, no real concordance in the scoring system across studies was disclosed. For this reason, a direct comparison of different studies could lead to misleading results. Furthermore, these series were published over several decades, with cases treated as late as in the ‘70s. It is therefore reasonable to assume that the increasing quality of treatment in recent years, especially for what concerns RT, might have influenced outcomes thus introducing other confounding factors in the comparison of these case series. Given these premises, some studies showed a statistical association between clinical outcome and EGFR overexpression [38-44], while other analyses failed to disclose such correlation [45-56]. A recent meta-analysis of 33 studies showed that although HNSCC patients with high EGFR expression had a poorer OS regardless of the type of treatment, a large heterogeneity was reported, mainly related to tumor site and IHC scoring system [57]. With respect to IHC, it is worth mentioning that when the studies were stratified according to the score systems, only the group that evaluated the staining based on a combination of intensity and extent showed significant association between EGFR expression and OS.

Two other studies investigated EGFR expression by IHC as a prognostic factor in patients treated with combination of CT and RT [58, 59]. They used several different chemotherapeutic agents as well as different scoring system, none of them relying on a quantitative approach. These studies failed in identifying EGFR overexpression as a prognostic factor. A positive study is represented by the work of Kumar et al [60], who prospectively evaluated EGFR expression, together with other markers, in advanced oropharyngeal SCC patients treated with one cycle of cisplatin or carboplatin and fluorouracil. Responders (i.e., those with 50% response at the primary site) received CT/RT; non-responders received surgery and RT. EGFR overexpression was associated with poor response to induction CT. Similar results were reported by Hitt et al [61].

The issue of EGFR expression deserves a further comment relative to oropharyngeal cancer, given the strong association between these tumor sites and the presence of HPV conferring a favorable prognosis. Several studies did not investigate HPV infection, therefore potentially altering the evaluation of the prognostic value of EGFR expression. An inverse correlation between HPV positivity and EGFR expression has been reported [22, 50,62-68], but the same studies reported discordant results on the prognostic value of EGFR expression.

Few trials [60, 63, 64, 67] evaluated the combined effect of HPV status and EGFR expression on prognosis, showing that the use of EGFR expression in combination with HPV status provides additional prognostic information. The prognosis of patients with EGFR-positive/HPV-negative cancer was the poorest, while the EGFR negative/HPV-positive group showed the best outcome. However, in recent studies on oropharyngeal HNC patients, the prognostic role of EGFR expression was related to the association with HPV-negative tumors, and the added value of EGFR analysis seems to be marginal in respect to HPV [22, 69].

### 1C) EGFR INHIBITORS

#### Prognosis

Some studies have investigated the relation between EGFR expression and the outcome of recurrent/metastatic HNSCC patients treated with anti-EGFR antibody cetuximab [36, 70-72]. Even if limited to restricted series, these investigations consistently showed that EGFR levels detected by IHC have no impact on response, DCR, PFS or OS of patients treated with cetuximab.

By contrast, when EGFR expression was assessed by IHC and real time PCR, a prognostic value for high EGFR expression has been associated with reduced PFS and complete response in cetuximab+RT-treated HNSCC patients, in two studies [73, 74].

However, in a phase II trial of induction CT with weekly cetuximab, paclitaxel, and carboplatin followed by chemoradiation in operable stage III/IV HNSCC, the identification of EGFR by a different approach, i.e. the quantitative AQUA method, confirmed the lack of prognostic value [75].

Last, the percentage of EGFR expression in the tumor cells was significantly associated with better OS in HNSCC patients receiving nimotuzumab in combination with CT [76], by contrast no association between EGFR expression and tumor outcome was observed in advanced HNSCC treated with nimotuzumab in combination with RT [77].

#### Prediction

In the EXTREME study, a trend to increased PFS and OS with cetuximab plus chemotherapy was observed for patients with higher IHC scores; however, the low number of patients does not allow any definitive conclusion [78]. The addition of cetuximab was able to counteract the dismal prognosis of the tumors with high EGFR expression showed by the group treated with chemotherapy alone.

Discordant results were obtained in Burtness' trial, in which patients were randomly assigned to receive cetuximab in combination with cisplatin or cisplatin alone [79]. Tumor samples were evaluated for EGFR cytoplasmatic expression by IHC and among the 52 patients categorized as EGFR low-to-moderate, the response rate was 41% for those treated with cisplatin plus cetuximab, compared with 12% for those treated with cisplatin and placebo (p=0.03). Thus, low-moderate EGFR expression seems to predict the response to cetuximab. However, in a logistic regression analysis of response, the interaction between EGFR and treatment group was found not to be significant. By contrast, Psyrri et al reported a correlation between EGFR expression (AQUA method) and response to cetuximab [36]. In a trial of concurrent chemoradiation ± cetuximab in advanced HNSCC, the analysis was not able to identify EGFR expression as a predictive biomarker because outcomes did not improve by adding cetuximab to RT-cisplatin and did not differ according to EGFR expression [72]. Considering treatment with nimotuzumab, a significant survival improvement was observed in EGFR positive unresectable HNSCC patients treated with this inhibitor and RT compared to control patients receiving placebo and RT [80].

It is worth mention the work by Del Campo *et al*. who investigated lapatinib in locally advanced HNSCC before chemo-radiotherapy. They reported that IHC EGFR overexpression seems to be predictive; however, the number of responding patients was too low to make any conclusions [81].

On the basis of the above-mentioned discordant data, EGFR expression is neither a prognostic nor a predictive factor in relation to EGFR inhibitors use.

## EGFR PROTEIN ACTIVATION

Whereas many studies investigated the role of total EGFR expression, only few focused on quantitative evaluation of activated receptor (Table 2), although phosphorylated EGFR (pEGFR) is thought to be a better biomarker for EGFR pathway activation. pEGFR can be assessed on frozen material by western blotting or reverse-phase protein and on fixed material by array fluorescence resonance energy transfer (FRET) or IHC.

**Table 2:** Main studies on EGFR protein activation as prognostic factor in HSCC

Study	Population	Treatment	EGFR assay	Prognostic value of EGFR activation
**Radiotherapy**				
Romanitan 2013	n=8, oropharyngeal SCC	Accelerated RT	IHC assay	No impact on OS, DFS
**Surgery, chemotherapy, radiotherapy**				
Wheeler 2012	n=67 frozen HNSCC	Surgery	Reverse-phase protein array	Impact on PFS
Hama 2009	n=82, frozen HNSCC	Surgery +/− CRT	Western blotting	Impact on relapse
Kong 2006	n=286 HNSCC	Surgery +/−RT	FRET	Impact on DFS
Szabo 2011	n=71, HNSCC	surgery	IHC assay	Impact on OS

HNSCC: head and neck squamous cell carcinoma; OS: overall survival; PFS: progression free survival; RT: radiotherapy: CRT: chemotherapy; IHC: immunohistochemistry; DFS: disease free survival; FRET: fluorescence resonance energy transfer

### 2A) RADIOTHERAPY

#### Prognosis

One recent case series investigated pEGFR expression via IHC on a population of oropharyngeal SCC treated in most cases with accelerated RT [82]. EGFR phosphorylation at residue Tyr1148 and Tyr1068 was detected in 20% and 47% of patients, respectively; no association between Tyr1148 or Tyr1068 activation and OS or DFS was observed. Interestingly, pEGFR Tyr1148 and Tyr1068 were associated with absence of HPV infection.

### 2B) SURGERY, CHEMOTHERAPY, RADIOTHERAPY

#### Prognosis

Wheeler and colleagues, assessing the phosphorylation specific sites Y992 and Y1068 using reverse-phase protein array, showed that intermediate or high tumor EGFR Y1068, but not Y992, phosphorylation were associated with significantly reduced PFS in a series of frozen HNSCC samples [73]. Notably, in the same series, intermediate/high EGFR Y1068 phosphorylation and intermediate/high EGFR expression were both independently associated with reduced PFS, thus representing potential prognostic indicators. Y1068 pEGFR was confirmed by western blotting in 17% of HNSCC patients who underwent surgery±CT where EGFR- phosphorylated tumors relapsed significantly earlier than not-phosphorylated ones [83].

Kong et al [84] investigated a large series of HNSCC via FRET, an automated technique able to inform about EGFR phosphorylation status. Of interest, EGFR activation did not correlate with EGFR expression, which in turn was not found to be prognostic.

Last, in a single isolated study, pEGFR assessed by IHC using a specific antibody targeting the phospho-tyrosine site 1086 was associated with prolonged survival [38].

### 2C) EGFR INHIBITORS

#### Predictivity

No predictive effect was observed for pre-treatment pEGFR level in patients with locally-advanced HNSCC receiving lapatinib before chemo-radiotherapy [81].

## EGFR GENE COPY NUMBER

In the studies reported in Table 3, EGFR gene status was assessed by FISH, and tumors showing high polysomy of chromosome or *EGFR* gene amplification were considered to have increased *EGFR* gene copy number and classified as FISH positive.

**Table 3:** Main studies on EGFR gene copy number as prognostic and predictive factor in HSCC

Study	Population	Treatment	EGFR assay	Prognostic value of EGFR gain	Predictive value of EGFR gain
**Radiotherapy**					
Ryott 2009	n=37, oral tongue SCC	Preoperative RT	FISH assay	No impact on pCR, OS	-
**Surgery, chemotherapy, radiotherapy**					
Chung 2006	n=75, HNSCC	Surgery or biopsy	FISH assay	Impact on PFS, OS	-
Temam 2007	n=134, HNSCC	Surgery	Quantitative real time PCR	Impact on DFS, OS	-
Nakata 2011	n=89, oral tongue SCC	Surgery	FISH assay	Impact on DFS, OS	-
Szabo 2011	n=71, HNSCC	Surgery	FISH assay	Impact on OS	-
Young 2011	n=240, HNSCC	CRT	FISH assay	Impact on FFS	-
Ryott 2009	n=65, oral tongue SCC	Primary RT;surgery +/− RT and CT	FISH assay	No impact on OS	-
Pectasides 2011	n=102, HNSCC	Primary RT; surgery +RT	FISH assay	No impact on OS	-
Wheeler 2012	n=154, HNSCC	Surgery +/− RT;+/− CRT	FISH assay	No impact on PFS	-
Huang 2012	n=160, oral cavity SCC	Surgery	FISH assay	No impact on OS, DFS	-
Dionysopulus 2013	n=253, larynx SCC	Surgery and/or RT	Real time PCR	No impact on OS, DFS	-
**EGFR inhibitors**					
Hitt 2012	n=29, recurrent and/or metastatic HNSCC	Paclitaxel + Cetuximab	FISH assay	No impact on response, OS, PFS	-
Argiris 2010	n=39, HNSCC	Docetaxel+cisplatin + Cetuximab	FISH assay	No impact on PFS, OS	-
Wheeler 2012	n=39, HNSCC	Docetaxel+cisplatin + Cetuximab+RT	FISH assay	No impact on PFS	-
Chau 2011	n=45 HNSCC	Erlotinib +cisplatin	FISH assay	No impact on response, TTP, OS	-
Cohen 2010	n=31, HNSCC	Primary CT + CRT + Gefitinib	FISH assay	Impact on OS	-
Licitra 2011	n= 312 recurrent and/or metastatic HNSCC	Platinum/5-FU +/− cetuximab	FISH assay	-	No association with response, PFS and OS

HNSCC: head and neck squamous cell carcinoma; OS: overall survival; PFS: progression free survival; RT: radiotherapy: CT: chemotherapy: CRT: chemotherapy; FISH: fluorescent in situ hybridization; FFS: failure free survival; pCR: pathological complete response; DFS: disease free survival; TTP: time to progression; -: the prognostic or predictive value of EGFR gene copy number was not investigated.

### 3A) RADIOTHERAPY

#### Prognosis

In a study on oral tongue SCC patients treated with preoperative RT, high *EGFR* gene copy number tended to be higher in patients who did not achieve a pathological complete response, even if statistical significance was not reached [23]. The lack of other data does not allow to reach any definite conclusion.

### 3B) SURGERY, CHEMOTHERAPY, RADIOTHERAPY

#### Prognosis

A number of studies have investigated the association between *EGFR* gene status and prognosis in HNSCC patients on different primary treatments, and overall discordant results were obtained.

In some studies, increased *EGFR* gene copy number was considered as a strong prognostic factor, significantly associated with shorter PFS and OS [38, 46, 65, 84-86].

Interestingly, in all these studies *EGFR* gene copy number did not correlate either with EGFR protein expression assessed by IHC or EGFR mRNA expression levels detected by microarray or RT-PCR. The fact that *EGFR* copy number status is a more reliable indicator than EGFR overexpression was confirmed by the observation that patients whose tumors co-exhibited increase of *EGFR* gene copy number and protein overexpression presented significantly shorter OS than patients with tumors negative for FISH status and positive for EGFR protein overexpression [46]. These results indicate that the alteration of *EGFR* copy number may not be related only to alterations of EGFR protein expression. Indeed *EGFR* copy number changes frequently occur in association with chromosome 7 aneusomy that confers worse survival rates. Therefore one possibility is that additional genes on chromosome 7 that are co-amplified with *EGFR* may be involved in promoting a more aggressive tumor behavior. In line with this hypothesis, in a series of 35 oral SCC patients, Gebhart found that a gain of short arm on chromosome 7 was linked to a higher rate of relapse and worse OS [87]. Alternatively, an increased *EGFR* copy number may be a surrogate marker of chromosomal instability, which confers an adverse prognosis in many tumors [88]. However, the mechanism by which EGFR FISH status contributes to the oncogenic effect in expressing cells remains unclear.

On the other hand, a number of studies reported lack of association between *EGFR* gene copy number and OS [23, 37, 40, 73, 89]. Of note, most of these studies also showed the correlation between *EGFR* gene copy number and EGFR protein expression levels. Thus, despite some studies are available, the actual prognostic value of the *EGFR* gene copy number remains controversial. However, when *EGFR* gene copy number was assessed by FISH, the different scores used to define the FISH-positive and negative cases could explain these inconsistent findings.

### 3C) EGFR INHIBITORS

#### Prognosis

The results obtained in different cohorts of locally-advanced or recurrent HNSCC patients treated with cetuximab-based regimens indicate that patients with FISH positive tumors do not show a better response compared with FISH-negative subjects [70] and that *EGFR* gene status is not associated with PFS or OS [70, 73, 90]. Furthermore, *EGFR* amplification was not associated with TTP or OS also in HNSCC patients treated in two phase II trials on erlotinib [91]. By contrast, EGFR FISH-positive status was associated with shorter OS in locally-advanced HNC patients receiving CT/RT and gefitinib [92].

Prediction

The phase III EXTREME study demonstrated that combining cetuximab with platinum-based CT significantly prolonged OS in the first-line treatment of patients with recurrent and/or metastatic HNSCC compared with CT alone. Samples deriving from patients receiving cetuximab plus CT or CT alone were investigated by EGFR FISH to evaluate the role of increased *EGFR* copy number as predictive biomarker [93]. No correlation between EGFR FISH score and response rate was observed in both study arms: therefore, the results of this unique-to-date study support the lack of utility of EGFR FISH status in predicting the response to cetuximab.

Furthermore, EGFR amplification showed no predictive role in patients treated with lapatinib in locally advanced HNSCC before chemio-radiotherapy [81].

## EGFR POLYMORPHISMS

### 4A) SURGERY, CHEMOTHERAPY, RADIOTHERAPY

EGFR polymorphisms were uniformly assessed by DNA PCR-based methods. A recent study reported the prognostic role for EGF and EGFR polymorphisms in a cohort of locally-advanced HNSCC patients treated with post-operative CT-RT (Table 4) [94]. In more details, the 5-year OS rates of patients with EGFR R521K G/G (11.1%) and G/A (15.9%) were lower than those for the A/A (62.5%) genotype. Patients carrying one or two unfavorable alleles had worse 5- year OS than those without unfavorable allele (not available versus 20% versus 71.4%, p=0.002). Multivariate analysis revealed that the highest risk of death was associated with the coexistence of two unfavorable genotypes (hazard ratio 25.7, 95% confidence interval =3.4–193.4; p=0.002).

The prognostic value of EGFR R521K polymorphism was further investigated in the study of Bandrés et al., where it was defined as R497K according to the older nomenclature [95]. Patients with G/G genotype of the polymorphism R521K in exon 13 showed the highest risk of disease-related mortality. In contrast, in the same patient cohort, the (CA)_n_ repeat polymorphism in intron 1 was not associated with OS. However, this single study does not allow any firm conclusion.

### 4B) EGFR INHIBITORS

#### Prognosis

Fifty-one recurrent/metastatic HNSCC patients enrolled in a single-arm, phase II study of second-line treatment with cetuximab/docetaxel were genotyped for EGFR polymorphisms R521K in exon 13 and (CA)_n_ repeat. The results revealed that R521K G/G genotype is significantly associated with increased skin toxicity, DCR and better PFS [96]. However, this genotype had no influence on OS.

However, in HNSCC patients treated with cetuximab±CT, the presence of R521K G/G genotype was associated with longer OS. In detail, the median OS was 6.7 months in patients with at least one K allele compared with 13.3 months in patients homozygous for the wild-type R allele [97].

The CA repeat polymorphism was not associated with DCR, PFS or OS [96].

At present, the promising prognostic value of EGFR R521K G/G polymorphism in patients treated with combination of surgery, CT and RT or EGFR inhibitors deserves further investigations. The predictive role of these polymorphisms has not been investigated yet.

## EGFR MUTATION

*EGFR* mutation is usually considered a rare event in HNSCC. However, the actual frequency of *EGFR* mutation is not well defined, even though analogous techniques, mainly PCR and sequencing, have been applied to investigate this issue [3, 98, 99].

### 5A) SURGERY, CHEMOTHERAPY, RADIOTHERAPY

#### Prognosis

Hama et al [83] prospectively analyzed a population of surgically-treated HNSCC (Table 4). Among 13 patients with EGFR phosphorylation, 4 with *EGFR* mutation had a longer survival without recurrence than patients with *EGFR* wild type (p=0.023). However, the mutational analysis performed on the largest number of cases (17 mutated cases out of 108 total cases) indicated that *EGFR* mutation is not a significant prognostic factor [99].

### 5B) EGFR INHIBITORS

#### Prognosis

A case report showed complete response in a patient with HNSCC treated with cetuximab monotherapy after initial surgery and RT. The authors identified a somatic mutations in the ligand-binding domain (P546S) and in the kinase domain (R705G) [100]. Interestingly, in *vitro* experiments indicated that the P546S mutation in the EGFR ligand-binding domain enhances NIH-3T3 cell line sensitivity to cetuximab compared with cells expressing wild-type *EGFR*; on the other hand, R705G mutation did not seem to contribute to drug sensitivity. In another study, a deletion in exon 19 of *EGFR* was disclosed in two out of 29 HNSCC patients treated with cetuximab and RT. These two patients presented poor clinical outcome, suggesting that this mutation may contribute to the limited response [74].

Despite this intriguing but overall weak evidence, it is rather unlikely that *EGFR* mutations could provide information about HNSCC response to anti EGFR therapy, mostly due to their low and variable rate in HNSCC.

## EGFRVIII EXPRESSION

### 6A) SURGERY, CHEMOTHERAPY, RADIOTHERAPY

#### Prognosis

EGFRvIII is a mutant form of EGFR due to a deletion of exons 2-7 resulting in a frame deletion variant with a truncated extracellular domain exerting ligand-independent constitutive activity.

Wheeler et al. investigated the expression levels of EGFRvIII mRNA, as detected by real time RT-PCR, in HNSCC patients treated with surgery and/or CT (Table 4) [73]. In this cohort of HPV-negative tumors, intermediate or high EGFRvIII mRNA levels did not provide any prognostic information.

Another study by Szabo et al evaluated EGFRvIII cell expression by IHC in a group of HNSCC patients [38]. Multivariate analysis showed no significant correlation between the presence of EGFRvIII and patient survival.

**Table 4:** Main studies on EGFR polymorphisms, mutation and EGFR VIII expression as prognostic and predictive factor in HSCC

**POLYMORPHISMS**				
Study	Population	Treatment	EGFR assay	Prognostic value
**Surgery, chemotherapy, radiotherapy**				
Nai-Wen Su 2014	n=180, HNSCC	Surgery +CRT	PCR and sequencing	Impact of EGFR R521K G/G and G/A on low OS
Bandrès 2007	n=67, HNSCC	Surgery and/or CRT	Fluorescent PCR; PCR-RFLP	Impact of EGFR R521K G/G on high DRM. No impact of (CA)n repeat polymorphism in intron 1 on OS.
**EGFR inhibitors**				
klinghammer 2010	n=51, HNSCC	Cetuximab+docetaxel	PCR-RFLP; PCR and sequencing	Impact of R521K G/G on DCR and better PFS. No impact of of R521K G/G and (CA)n on OS.
Stoehlmacher-Williams J 2012	n=48, HNSCC	Cetuximab +/CT	PCR-based RFLP	Impact of R521K G/G on longer OS
**MUTATION**				
**Surgery, chemotherapy, radiotherapy**				
Hama 2009	n=82, HNSCC	Surgery +/− CRT	PCR and sequencing	Impact on longer survival without recurrence
Na 2007	n=108, tongue and tonsil SCC	Surgery and or RT	PCR and sequencing	No impact on OS
**EGFR inhibitors**				
Bahassi 2013	Case report	Surgery and RT followed by cetuximab	PCR and sequencing	Impact on response
Smilek 2012	n=29, HNSCC	RT + cetuximab	Real time PCR	Impact on response
**EGFR VIII EXPRESSION**				
**Surgery**				
Wheeler 2012	n=49, HNSCC	surgery	Quantitative real time PCR	No impact on PFS
Szabo et al.	n=71, HNSCC	surgery	IHC	No impact on OS
**EGFR inhibitors**				
Tinhofer 2011	n=45, HNSCC	docetaxel+cetuximab	IHC	impact on treatment response and PFS
Chau 2011	n=53, HNSCC	erlotinib+cisplatin	Real time PCR	Impact on better DCR
Smilek	n=29, HNSCC	cetuximab +rt	Real time PCR	No impact on treatment response
**EGFR LIGAND EXPRESSION**				
**Radiotherapy**				
Aebersold 2002	n=95, oropharyngeal SCC	curative rt	IHC	No impact of TGFα on prognosis
Wen 1996	n=68 laryngeal SCC	rt	IHC	Impact of TGFα on recurrence
Surgery				
Rubin 1998	n=91, HNSCC	surgery+/−rt	IHC	Impact on DFS, cause specific survival
**EGFR inhibitors**				
Tinhofer 2011	n=47, HNSCC	cetuximab+docetaxel	IHC	Impact of amphiregulin on PFS, OS

HNSCC: head and neck squamous cell carcinoma; OS: overall survival; PFS: progression free survival; DRM: disease related mortality; DFS: disease free survival; DCR: disease control rate; RT: radiotherapy: CT: chemotherapy: IHC: immunohistochemistry; PCR: polymerase chain reaction; RFLP: restriction fragment length polymorphism

### 6B) EGFR INHIBITORS

#### Prognosis

Biopsies from patients enrolled in a single-arm phase II study investigating cetuximab plus docetaxel as second-line treatment of recurrent/metastatic HNSCC were analyzed by IHC to measure EGFRVIII expression. EGFRvIII expression was detected in 17% of cases and there was a significant association between EGFRvIII levels and treatment efficacy. In the group of patients with low EGFRvIII IHC score, DCR was 65% whereas patients with high EGFRvIII score showed a DCR of 13% (p=0.02). Multivariate logistic regression analysis confirmed the independent association of EGFRvIII with lack of response and shorter PFS [71]. Moreover, EGFRvIII level, detected by real time PCR, was associated with increased DCR but not with time to progression or OS in recurrent or metastatic HNSCC patients treated with erlotinib [91]. However, Smilek et al reported no association between RT-PCR detected EGFRvIII levels and response to cetuximab combined with RT [74].

It must be emphasized that the presence of EGFRvIII in HNSCC reported in the mentioned studies may be questioned. In fact, two studies recently analyzed a large number of tumors (638 and 531 HNSCC samples) for EGFRvIII expression using IHC with the antibody L8A4, RT-PCR and the RNA-Seq analysis [101, 102]. Both studies provided a strong evidence showing that EGFRvIII is absent or very rare (frequency of 0.37%) in HNSCC. In any case, when present, EGFRvIII expression level is low in HNSCC [103] and thus difficult to detect. Cumulatively, the different methodologies applied, coupled with the intratumoral heterogeneity, may explain the discordant results and support the notion that an accurate detection of EGFRvIII needs multiple methodologies including DNA, RNA and protein assessment [103].

## EGFR LIGAND EXPRESSION

### 7A) RADIOTHERAPY

#### Prognosis

In a group of patients with oropharyngeal cancer who underwent curative RT, the levels of the EGFR ligand TGFα assessed by IHC in pretreatment tumor biopsies was not a prognostic marker (Table 4) [21]. By contrast, in a group of 68 patients with early laryngeal cancer treated with RT, the recurrence rate was significantly higher in patients with tumor showing TGFα expression detected by IHC [19].

### 7B) SURGERY, CHEMOTERAPY, RADIOTHERAPY

#### Prognosis

TGFα levels were quantified by IHC and computerized image analysis system on primary tumors from HNSCC patients surgically treated (±RT-CT). At multivariate analysis, high TGFα levels were predictors of reduced DFS and disease-related mortality [35].

### 7C) EGFR INHIBITORS

#### Prognosis

Expression of the EGFR ligand amphiregulin was assessed by IHC on biopsies from recurrent/metastatic HNSCC patients treated with cetuximab plus docetaxel. A trend towards a reduction in DCR (40%) was observed (p=0.09) in patients with high amphiregulin IHC score, as compared with patients with low score (65%) [71]. In addition, patients that showed high expression of amphiregulin had significantly shorter PFS and OS. Due to the paucity of data, no firm conclusion can be made about the prognostic role of EGFR ligands.

**Table 5:** Overview of Authors' conclusions.

	EGFR													
	expression		activation		copy number		polimorphisms		mutation		EGFRvIII		ligands	
	prog	pred	prog	pred	prog	pred	prog	pred	prog	pred	prog	pred	prog	pred
RT	yes	yes (accelerated RT)	no	n.i.	no	n.i.	n.i.	n.i.	n.i.	n.i.	n.i.	n.i.	d.r. TGFα	n.i.
Surgery-CT-RT	yes	n.i.	yes*	n.i.	d.r.	n.i.	yes R521K*	n.i.	no	n.i.	d.r.	n.i.	yes TGFα*	n.i.
EGFR inhibitors	d.r.	no	n.i.	n.i.	no	no	yes R521K*	n.i.	weak evidence	n.i.	d.r.	n.i.	yes amphiregulin*	n.i.

prog: prognostic; pred: predictive; CT: chemotherapy; RT: radiotherapy; d.r.: discordant results; n.i.: not investigated;

* indicative results to be validated

## CONCLUSION

We reviewed the literature on the clinical significance of EGFR alterations in relation to the HNSCC treatment, with the aim to identify emerging prognostic or predictive factors. Our conclusions are summarized in Table 5.

The majority of the trials explored the prognostic role of EGFR alterations, while only limited data exist on the predictive role of EGFR alterations in relation to a specific treatment. At present, the predictive role of this marker has been exclusively explored in HNSCC patients treated with RT or EGFR inhibitors.

Concerning RT, high EGFR expression is a negative prognostic factor associated with poor outcome. Convincing evidence supports the robust predictive role of high EGFR expression when evaluated by a quantitative assay, in order to select patients who can most benefit of accelerated RT.

For HNSCC patients receiving multiple treatments such as surgery, CT and RT or a combination of them, no predictive biomarkers are available. However, several studies indicated that EGFR expression represents a good prognostic parameter, even if there is heterogeneity mainly due to IHC scoring system and tumor site variability. Notably, the significant association of high EGFR expression with shorter PFS and OS was reproducible only when protein expression was measured by a “quantitative” or at least semi-quantitative method. It appears advisable to estimate EGFR protein by a IHC method considering both intensity and extent of the staining, as well as stratifying patients by specific tumor sites. Nuclear EGFR and TGFα levels seem to be additional promising negative prognostic factors, as well as EGFR activation and polymorphisms, however they have not been widely validated yet. *EGFR* gene copy number as well as EGFR mutations has no prognostic value and do not deserve further investigation.

Within the field of EGFR inhibitors, mainly cetuximab, available studies established that both EGFR expression and an increased gene copy number are neither predictive nor prognostic biomarkers. EGFR polymorphisms and high amphiregulin levels may be promising prognostic factors and validation trials are necessary. However, it must be taken into account that the immune system plays a role in the clinical response to the EGFR inhibitor cetuximab too. Indeed, in addition to the inhibition of downstream signaling pathways, cetuximab mediates its effects also by immunogenic mechanisms such as the antibody-dependent cell mediate cytotoxicity (ADCC), the complement-mediate cytotoxicity, the modulation of the human leukocyte antigen class I and antigen-processing machinery component expression [104]. This fact should be taken into account also considering the emerging role of checkpoint inhibitors in head and neck cancer and the possible synergistic effect of cetuximab and immunotherapeutic drug combinations.

Moreover, when interpreting these data, the inverse relationship between EGFR expression or *EGFR* increased gene copy number and the stronger prognostic factor of HPV positivity should be taken into account. HPV-positive oropharyngeal tumors tend to present decreased EGFR expression [50, 60, 63, 64, 67, 105], and increased EGFR gene copy number is restricted to HPV-negative cancer [3, 65]. Although the rationale underlying those findings has not been fully elucidated yet, it is widely accepted that HPV-positive tumors have a different genetic profile compared with HPV-negative counterparts, thus contributing to the different clinical behavior and to the higher chemo- and radiosensitivity [68, 105, 106]. Therefore, the greater impact of EGFR deregulation in HPV negative tumors should be analyzed within the context of this different genetic pattern, and the “combined” effect of HPV status and EGFR expression on prognosis both of HPV-positive and negative or only of HPV-negative patients remains to be defined.

Lastly, it should be stressed the importance to increase the research regarding the molecular mechanisms underlying EGFR deregulation, in order to improve the HNSCC therapeutic approaches and to reduce the discrepancy sometimes existing between preclinical and clinical data.
